# Neuroepithelial tumors of the central nervous system with *EWSR1::PATZ1* fusion: a case report and literature review

**DOI:** 10.3389/fonc.2025.1604479

**Published:** 2025-06-12

**Authors:** João Felipe Lima Feldmann, João Henrique Lima Feldmann, Felipe Sales Canedo, Felipe Cicci Farinha Restini, Romulo Loss Mattedi, Luiz Guilherme Cernaglia A. de Lima, Olavo Feher

**Affiliations:** ^1^ Clinical Oncology Department, Hospital Sírio-Libanês, São Paulo, SP, Brazil; ^2^ Radiotherapy Department, McGill University Health Center, Montreal, QC, Canada; ^3^ Pathology Department, Hospital Sírio-Libanês, São Paulo, SP, Brazil

**Keywords:** *EWSR1::PATZ1* fusion, *PATZ1* tumors, neuroepithelial tumor, WHO classification, next-generation sequencing

## Abstract

Neuroepithelial tumors (NEpT) harboring *EWSR::PATZ1* fusions remain an enigma. Initially described in sarcomas, these tumors display remarkable histomorphological diversity and unpredictable clinical behavior based on histologic or molecular features, with no established management protocols. To date, this subgroup of neoplasms has not been acknowledged as a *sui generis* entity by the WHO classification system, and it is currently designated as ‘NEC’/’NOS’. This retrospective case series describes two young adults (32–35 years old) without cancer predisposition or risk factors, diagnosed with *EWSR1::PATZ1*-fused NEpT. Case 1, a female with seizures, presented a heterogeneous left parietal lobe lesion (4.0 × 3.2 × 3.6 cm), classified as high-grade NEpT with *MGMT* promoter methylation, a calibrated score of 0.95 (≥ 0.9), and a co-occurring somatic *MUTYH* mutation. Case 2, a male with chronic headaches and mild right-sided paresthesia, had a left frontotemporal lesion (3.0 × 2.8 × 3.4 cm), initially diagnosed as an extraventricular neurocytoma but later reclassified as a NEpT with low-to-intermediate grade features, without MGMT methylation, and a calibrated score of 0.92. Case 1 received upfront resection, followed by Stupp protocol chemoradiation and temozolomide maintenance, resulting in 14 months of progression-free survival (PFS). Case 2 underwent subtotal resection and adjuvant radiotherapy after an 8-month recurrence, achieving 11 months of PFS to date. Both patients are asymptomatic, off corticosteroids, with the latest imaging revealing no disease progression. Our cases emphasize that *EWSR1::PATZ1*-fused NEpT displays a unique signature (ventricular localization, glioneuronal differentiation, and a distinct methylation cluster), supporting their inclusion in the WHO classification. Moreover, we present the first documented somatic co-mutation involving *MUTYH*. At present, despite the theoretical risk of temozolomide resistance due to *PATZ1* overexpression, our results suggest that conventional glioma therapies remain the preferred approach.

## 1 Introduction

The classification of central nervous system (CNS) tumors is evolving rapidly, driven by advances in molecular pathology and the widespread adoption of next-generation sequencing (NGS). The 2021 WHO Classification integrated molecular criteria into diagnostics, expanding the spectrum to over 100 distinct primary brain tumors (1). Although this shift improved diagnostic precision, it has unmasked previously unknown genetic alterations, now categorized as *not otherwise* sp*ecified* (NOS) or *not elsewhere classified* (NEC), whose clinical implications remain under investigation. Epidemiological data from the Central Brain Tumor Registry of the United States (CBTRUS) estimate that these categories account for approximately 11% of gliomas. However, this prevalence is considered provisional and is expected to decline as the implementation of molecular techniques enables reclassification into distinct subtypes (2).

The inherent vulnerability of the EWSR1 gene to chromosomal breakage and translocation often results in its involvement in fusion events with different molecular partners, contributing to the development of diverse cancers (7). The *EWSR1::PATZ1* gene fusion was initially described in sarcomas, with only four published series documenting approximately 30 cases to date. Most reported tumors arise in the thoracoabdominal region, exhibit round or spindle-cell low-grade or high-grade morphology, demonstrate polyphenotypic (ambiguous – neuro and muscular, specially rhabdomyoblastic) immunoprofile, and mainly display aggressive clinical behavior with poor response to conventional chemotherapy (3).

Since its initial identification in a CNS tumor in 2016, the *EWSR1::PATZ1* fusion has been reported across diverse primary CNS tumors, including NEpT, ependymoma, ganglioglioma, pleomorphic xanthoastrocytoma, low-grade glial/glioneuronal tumors, astroblastoma and glioblastoma (5). Nonetheless, consensus on the optimal diagnostic criteria or management strategies for these tumors remains elusive, underscoring a critical gap in the literature. *PATZ1* fusion-driven tumors are associated with upregulated anti-apoptotic pathways, and elucidating these mechanisms is critical for the development of novel strategies (4). Herein, we present two rare cases of primary CNS NEpTs harboring *EWSR1::PATZ1* fusions, a unique clinical course, and co-existing previously undescribed genetic and methylation alterations in this tumor type.

## 2 Case report and results

### 2.1 Case 1

**Figure 1:**
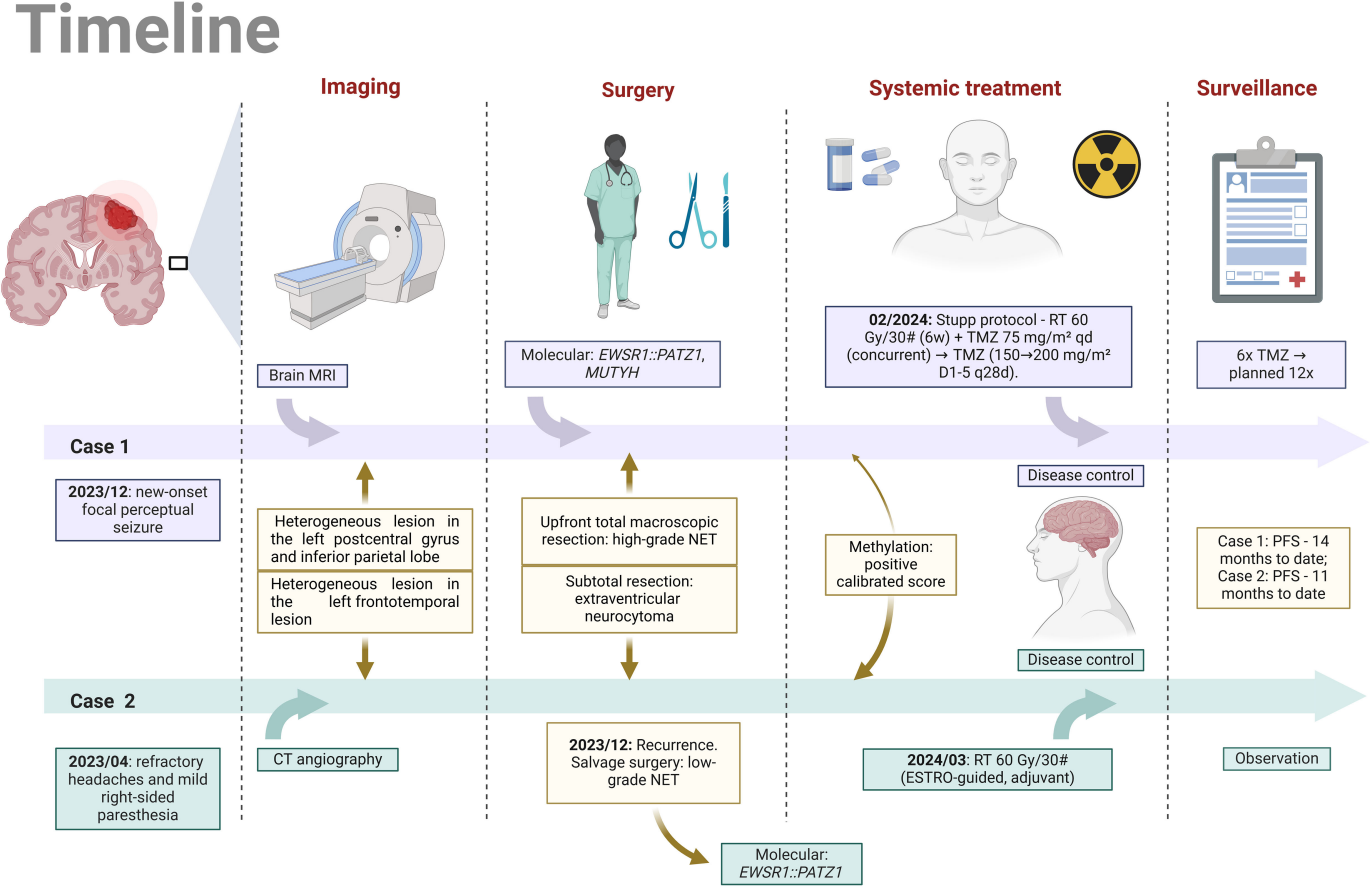
Parallel timeline of the clinical histories of Case 1 and Case 2 (Image created by the author, 2025). MRI, magnetic resonance imaging; CT angiography, computed tomography angiography; NET, neuroepithelial tumor; RT, radiotherapy; Gy, Gray; TMZ, temozolomide; PFS, progression-free survival; D1-5, q28d, administered on days 1 to 5 of a 28-day cycle.

**Figure 2:**
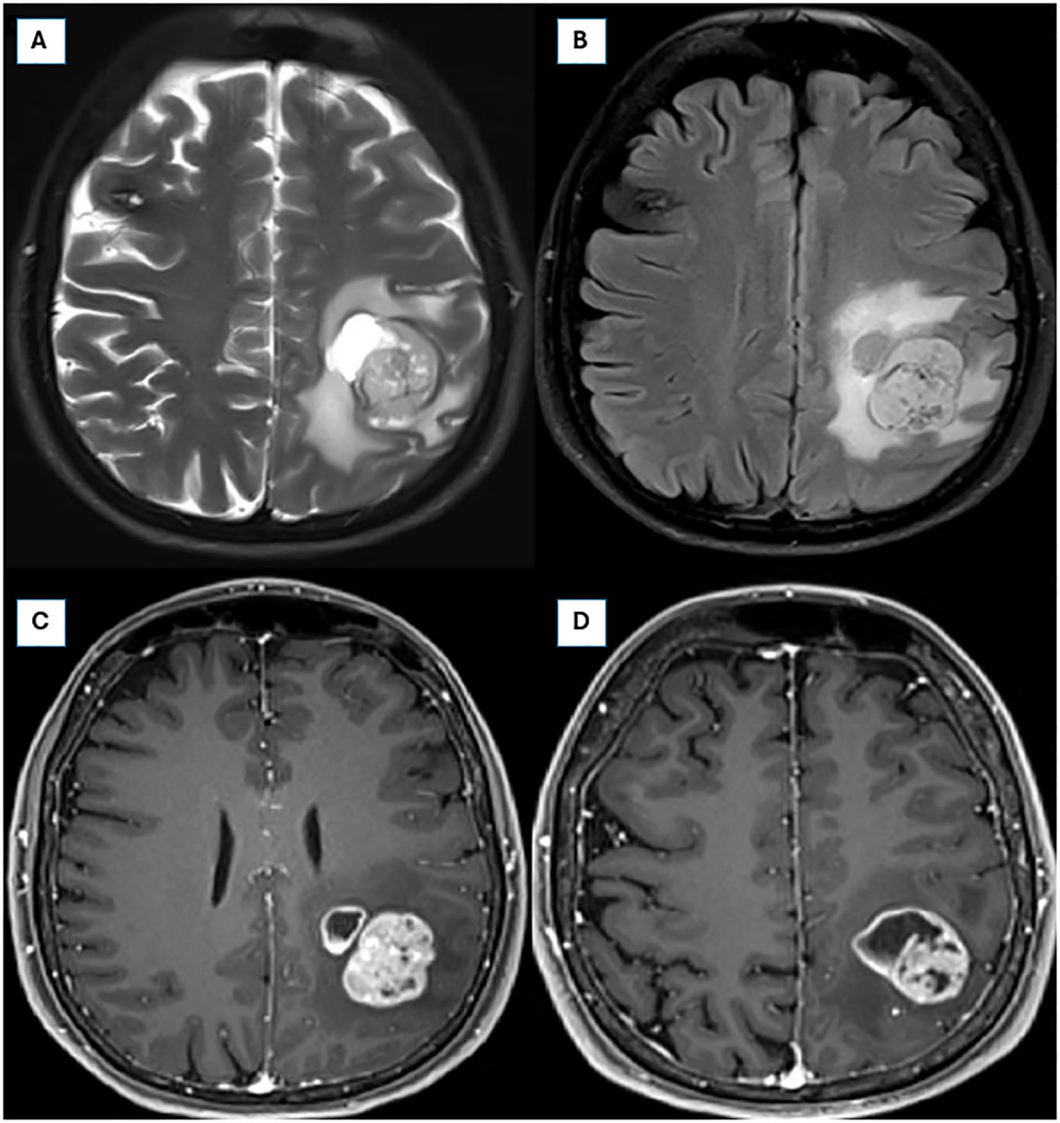
Preoperative brain magnetic resonance imaging (MRI) from 01/04/2024. **(A, B)** Axial T2 fat-saturated and FLAIR sequences, respectively, illustrating an infiltrative expansile lesion in the left parietal lobe, with cystic and solid components, prominent necrosis, and perilesional vasogenic edema, measuring approximately 4.2 × 3.8 × 2.6 cm. **(C, D)** Axial post-contrast T1-weighted sequences demonstrating intense and heterogeneous contrast enhancement.

**Figure 3:**
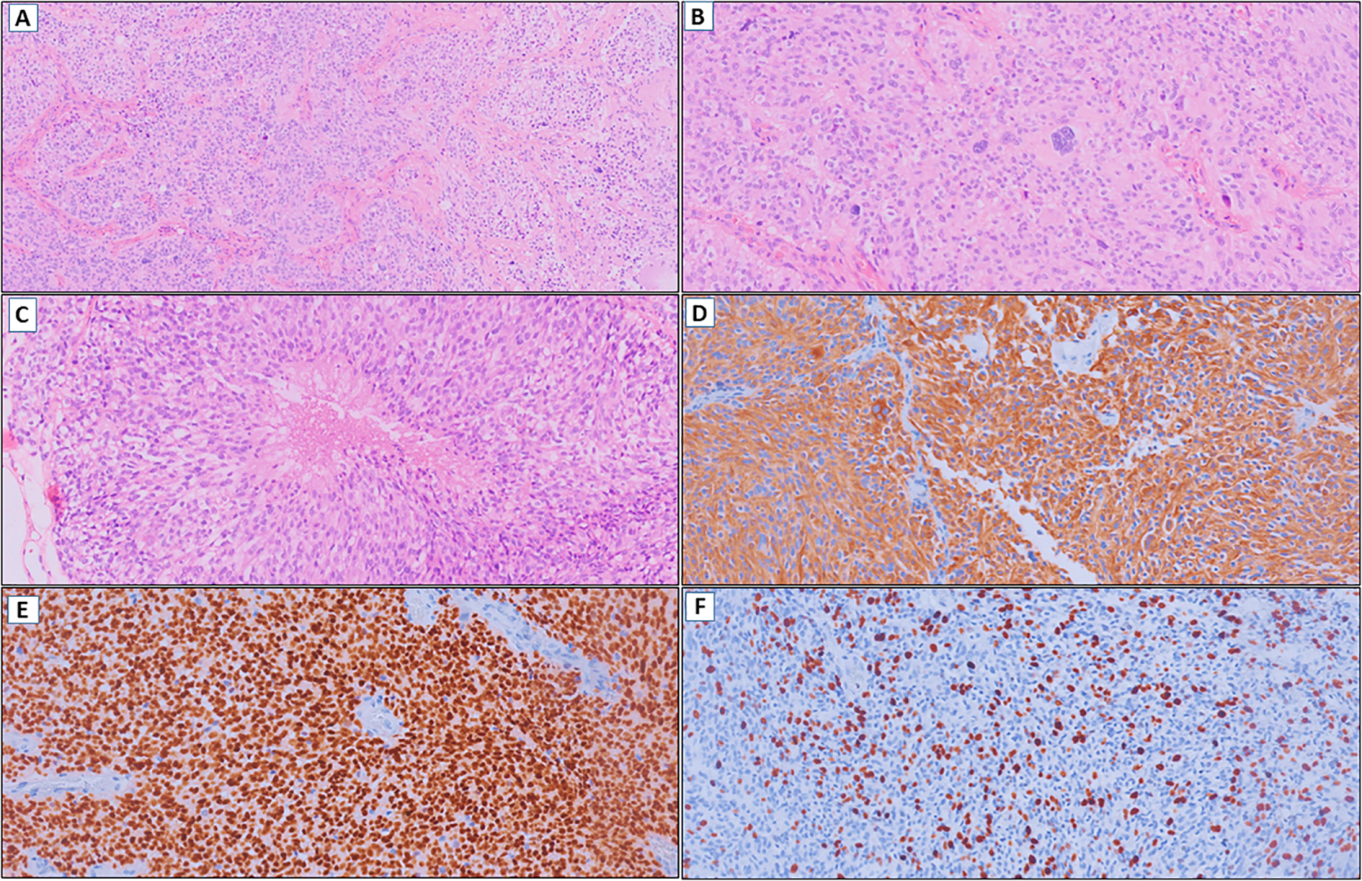
**(A)** Glioma with hypercellularity and vascular proliferation (HE, 100x). **(B)** Cellular pleomorphism (HE, 200x). **(C)** Tumor necrosis (HE, 200x). **(D)** Diffuse immunoexpression of GFAP (IHC, 200x). **(E)** OLIG2 positive (IHC, 200x). **(F)** Ki-67 with high cell proliferation index (IHC, 200x).

A 35-year-old right-handed woman with no significant medical, psychosocial or family history presented with a new-onset focal perceptual seizure, described as difficulty to brush her teeth and aphasia, lasting 2 minutes (see **Figure 1** for timeline). Brain magnetic resonance imaging (MRI) showed a 4.0 x 3.2 x 3.6 cm heterogeneous lesion in the left postcentral gyrus and inferior parietal lobe, as shown in **Figure 2**. Complementary studies with proton spectroscopy revealed decreased N-acetylaspartate and increased choline peaks, while perfusion imaging showed elevated relative cerebral blood volume (rCBV), suggesting glioma. She underwent gross total resection, and histopathological analysis confirmed a high-grade NEpT with a glial immunophenotype, characterized by hypercellularity, vascular proliferation, cellular pleomorphism, and tumor necrosis (**Figures 3A–C**). Immunohistochemistry revealed diffuse GFAP and OLIG2 positivity (**Figures 3D, E**), a Ki-67 proliferative index of 70% (**Figure 3F**), focal L1CAM expression, retained ATRX nuclear staining, and absence of IDH1 R132H mutation. At this point, differential diagnoses included diffuse glioma with non-canonical *IDH1/2* alterations, neuroepitelial tumors with *EP300-BCOR* fusion, astroblastoma *MN1*-altered and so-called “anaplastic” ependymoma/with histological anaplastic features, warranting molecular evaluation.

Molecular analysis was performed using DNA and RNA NGS from an in-house validated comercial panel. Test specifications and technical parameters are detailed in the Methods section. NGS testing identified an *EWSR1* (NM_005243.4) exon 8::*PATZ1* (NM_014323.3) exon 1 fusion (chimeric transcript – 1365 reads) and a heterozygous *MUTYH* mutation (c.1103G>A, p.Gly368Asp), inferred from a mutant allele frequency (MAF) of 6.3% and coverage of 627 reads. Somatic/germline origin could not be definitively resolved. NGS also identified five variants of uncertain significance: FUBP1: c.243A>C, p.Gln81His; JAK2: c.2765G>A, p.Arg922Gln; MRE11: c.1667A>G, p.Asn556Ser; PGR: c.1984G>A, p.Val662Ile; and TET2: c.356A>G, p.Asn119Ser. The diagnosis of CNS neuroepithelial tumor with *EWSR1::PATZ1* fusion was confirmed through tumor methylation profiling. This analysis revealed a methylated *MGMT* gene promoter, non-flat copy number variation (CNV) plot characterized by chromosomal gains in 1q, 5, 7, and 21, chromosomal losses in 1p, 6, 9p, 11, 15q, 17p, and 22q, as well as the absence of gene amplifications and homozygous deletion of *CDKN2A/B*, with a calibrated score of 0.95 for the diagnosis. Additionally, the tumor exhibited microsatellite stability and a low mutational burden (0 Mut/Mb) based on NGS panel.

Due to the lack of robust data to guide specific treatments for NEpT, the patient underwent standard radiotherapy treatment with 6 weeks of external beam radiation with a total dose of 60 Gy combined with concomitant temozolomide at a dose of 75 mg/m²/day. This was followed by 6 cycles of maintenance temozolomide (cycle 1 at 150 mg/m², with dose escalation to 200 mg/m² in cycles 2-6), administered for 5 consecutive days every 28 days, in accordance with the Stupp et al. protocol. The patient is scheduled to continue treatment for a total of 12 cycles. As of the most recent clinical assessment, after the 6th cycle of maintenance temozolomide, the patient remained asymptomatic, with excellent treatment tolerance, no new neurological deficits, and was on regular lacosamide use, without the need for corticosteroids. The last MRI demonstrated stable enhancing foci in the surgical bed, and there were no new abnormal enhancing foci or other concerning alterations.

### 2.2 Case 2

A 32-year-old left-handed man, with no significant comorbidities or psychosocial issues and a limited family history of cancer (in second-degree relatives), presented to the Oncology Department with a left frontotemporal lesion (3.0 × 2.8 × 3.4 cm) identified on computed tomography (CT) angiography. The lesion was suspected to be a primary CNS tumor and was discovered during the evaluation of a year-long history of refractory headaches and mild right-sided paresthesia. A subtotal resection was performed, leaving a residual mass of 1.8 × 1.8 cm. The outside pathology report suggested a diagnosis of extraventricular neurocytoma. The immediate postoperative period was complicated by a minor surgical wound infection, which was effectively treated with a short course of broad-spectrum antibiotics. The patient then began regular clinical follow-up.

**Figure 4:**
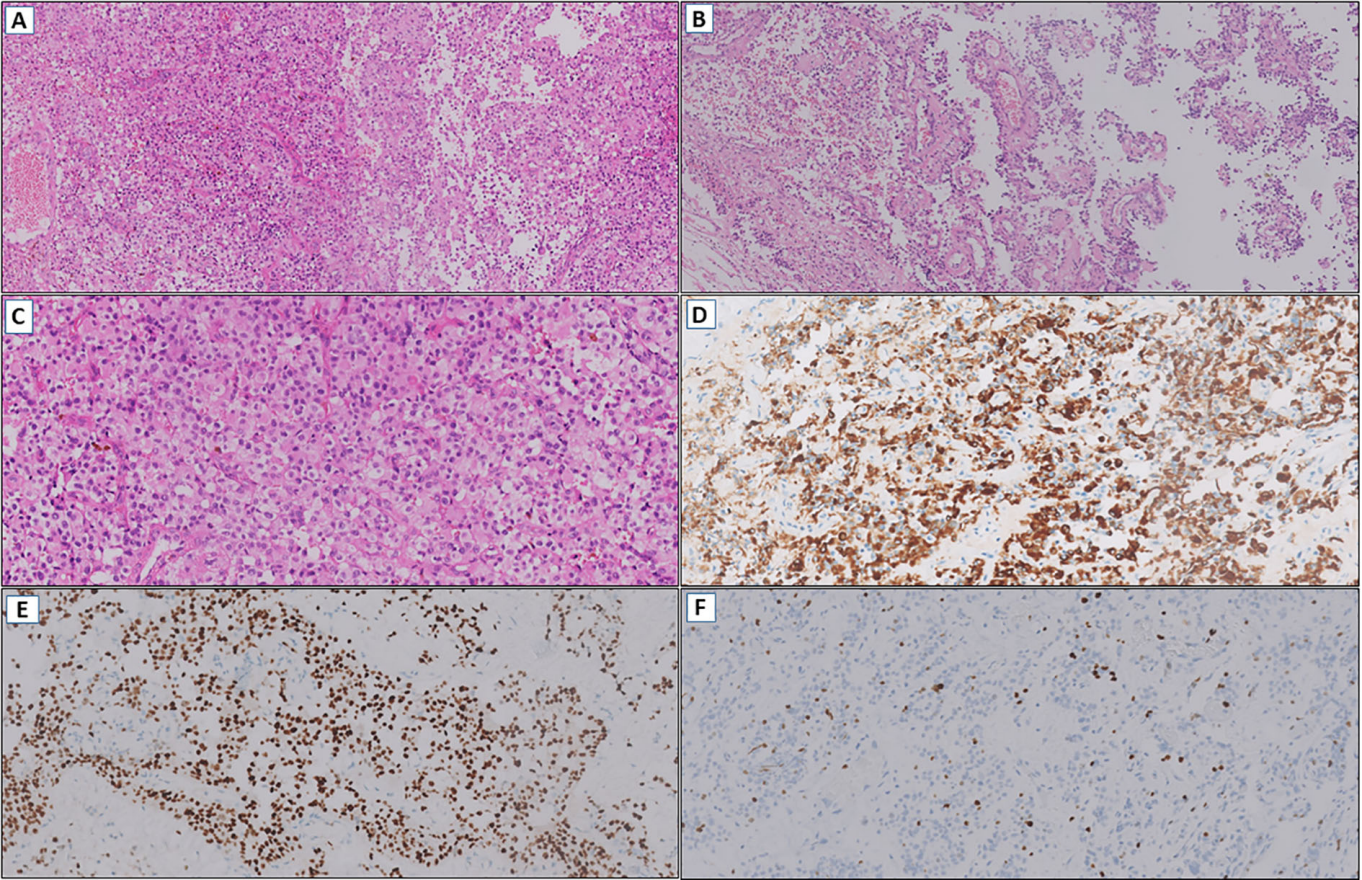
**(A)** Glioma with moderate cellularity (HE, 100x). **(B)** Perivascular growth pattern (HE, 100x). **(C)** Tumor cells with large eosinophilic cytoplasm (HE, 200x). **(D)** Diffuse immunoexpression of GFAP (IHC, 200x). **(E)** OLIG2 positive (IHC, 200x). **(F)** Low cell proliferation index by Ki-67 (IHC, 200x).

Eight months later, an MRI revealed an increase in the size of a heterogeneous nodular mass in the left subcentral region, with necrotic and cystic features, measuring approximately 2.0 × 1.5 cm. A slide review of the initial diagnosis was performed by two pathologists experienced in CNS tumors, with an impression of a primary tumor with glial differentiation, characterized by moderate cellularity, perivascular growth pattern, and cells with large eosinophilic cytoplasm (**Figures 4A–C**). Immunohistochemistry demonstrated strong GFAP and OLIG2 expression (**Figures 4D, E**), a Ki-67 proliferation index of 2-3% (**Figure 4F**), absent IDH1 R132H immunoreactivity, and retained ATRX expression. At the same time, after a multidisciplinary discussion, the team decided on a new surgical approach for local control and molecular profiling, with approximately 80% of the residual mass being resected. The pathology report of this surgical specimen matched the slide review.

Extended molecular analysis by NGS using the same in-house validated DNA and RNA comercial panel confirmed the presence of the *EWSR1* (NM_005243.4) exon 8::*PATZ1* (NM_014323.3) exon 1 fusion (chimeric transcript - 137 reads). Additional variants of uncertain significance included: APC: c.278_280delinsACG, p.Leu93_Arg94delinsHisGly and c.280C>G, p.Arg94Gly; ATR: c.4351C>T, p.Arg1451Trp; BLM: c.3970C>T, p.His1324Tyr; DNMT1: c.3458_3466dup, p.Pro1153_Leu1155dup; EPHA3: c.919G>A, p.Glu307Lys; FANCD2: c.3278T>A, p.Val1093Asp; MDM2: c.59T>G, p.Val20Gly; MYB: c.1447A>C, p.Lys483Gln; and PMS1: c.1615A>G, p.Met539Val. The diagnosis of a CNS neuroepithelial tumor with *EWSR1::PATZ1* fusion was established through an integrated approach with methylation profiling. This analysis revealed absent *MGMT* gene promoter methylation, along with small chromosomal losses in 1p, 3, 6, 9, 10, 11, 13q, and 20p. No gene amplifications or homozygous deletions of *CDKN2A/B* were detected, with a calibrated score of 0.91 for the diagnosis. Additionally, the tumor exhibited microsatellite stability and a low mutational burden (1 Mut/Mb) based on NGS findings. Comparative methylation profiling results are summarized in **Table 1**.

**Table 1:** Comparative methylation profiling of both cases.

DNA Methylation Profiling	Case 1	Case 2
Methylation Class	Neuroepithelial tumor with PATZ1 fusion	Neuroepithelial tumor with PATZ1 fusion
Calibrated Score	0.95	0.91
MGMT Promoter Methylation Status	Methylated	Unmethylated
Chromosomal Losses	1p, 6, 9p, 11, 15q, 17p, and 22q	1p, 3, 6, 9, 10, 11, 13q, and 20p
Chromosomal Gains	1q, 5, 7, and 21	NR
Gene Amplifications	NR	NR
CDKN2A/2B Homozygous Deletion	NR	NR

MGMT (O⁶-methylguanine-DNA methyltransferase), NR (Not reported).

Adjuvant radiotherapy was administered to the surgical cavity with clinical margins as per the European Society for Radiotherapy and Oncology (ESTRO) guideline with a total dose of 60 Gy in 30 fractions. Two months later, the patient experienced a generalized tonic-clonic seizure that lasted 1 minute, with no associated changes in imaging. At the most recent clinical evaluation, ten months after treatment completion, the patient remained asymptomatic, fully functional, and was on regular lacosamide use without the need for corticosteroids. The latest brain MRI showed stable enhancing foci in the surgical bed, with no additional abnormal findings in other areas. Clinical follow-up, including imaging and neurological examination, is planned every 3 months.

## 3 Methods

### 3.1 Histopathological and immunohistochemistry

Histopathological evaluation was performed on formalin-fixed paraffin-embedded (FFPE) tissues: Case 1 (6 blocks from surgical resection of a parieto-occipital tumor) and Case 2 (4 blocks from a left frontal biopsy). Tissue fixation was conducted in 10% neutral buffered formalin. Tissue sections were stained with hematoxylin and eosin (H&E). Immunohistochemistry (IHC) was conducted using a polymer-based detection system following deparaffinization and antigen retrieval with laboratory-specific protocols (Bacchi Laboratory for Case 1; Hospital Sírio-Libanês for Case 2). All antibodies used were clinically validated with internal positive and negative controls for each assay. The antibody panel included GFAP (polyclonal, Dako [Case 1]; clone EP672Y, Cell Marque [Case 2]), OLIG2 (clone Olg2, Sigma-Aldrich [Case 1]; clone EP112, BioSB [Case 2]), IDH1 R132H (clone H09, Dianova), ATRX (clone AX1, Sigma-Aldrich [Case 1]; polyclonal, Sigma-Aldrich [Case 2]), L1CAM (clone 14.10, Leica), Ki-67 (clone MIB1, Dako), and H3K27me3. Case 1 assessed mismatch repair proteins (PMS2 [clone EPS1], MLH1 [clone ES05], MSH2 [clone G219-1129], MSH6 [clone EP49]). Case 2 additionally assessed Synaptophysin (clone SP11, Ventana), Neurofilament (clone 2F11, Cell Marque), and EMA/TTF-1/AE1-AE3. Positivity thresholds were defined as ≥5% tumor cells (except Ki-67, which was visually quantified as a percentage). Results were independently reviewed by board-certified neuropathologists in accordance with the 2021 WHO CNS classification.

### 3.2 Next generation sequencing

DNA and RNA were extracted from FFPE tumor tissue. NGS was performed using the Illumina TruSight™ Oncology 500 (TSO500) panel, targeting 510 genes for SNVs, MNVs, indels (≤30 bp), and CNVs (59 genes), as well as RNA-based detection of gene fusions (55 genes) and alternative isoforms (3 genes). Sequencing was carried out on the Illumina^®^ NextSeq 550 platform, with data analysis via the TSO500 Local App (v2.2.0) and BaseSpace Variant Interpreter (v3.6.2.0), incorporating a custom bioinformatics pipeline. Variants were filtered at MAF ≥5% and coverage ≥100×. Tumor mutational burden (TMB) and microsatellite instability (MSI) were assessed using Illumina-validated algorithms; MSI results were considered unreliable when fewer than 20% of loci were unstable. Average sequencing depth after UMI deduplication was 1,607×, with 99.0% of exonic regions covered at >500×. CNV thresholds were predefined (e.g., ≥8 copies for EGFR, ERBB2, FGFR3, MET, MYC, MYCN; ≥10 copies for others), and no amplifications were detected. Due to suboptimal TERT promoter coverage, a complementary TagMan PCR assay was used to rule out the c.-124C>T mutation. Assay validation demonstrated high analytical performance: for SNVs, sensitivity ranged from 82.9% (MAF 2.5%) to 98.2% (MAF 10%); for indels, 76.4% to 100%. Specificity for both SNVs and indels was 100%, with PPV >99%. Intra- and inter-assay reproducibility were 98.9% and 100%, respectively. Accuracy per alteration type was: SNVs (98.7%), indels (97.9%), CNVs and fusions (100%), MSI (95.5%), and TMB (Pearson r = 0.9, p < 0.001). Internal clinical controls were included in all runs. Reference databases included GRCh37.p13, RefSeq v105, COSMIC v89, ClinVar (20191105), and gnomAD r2.1.

### 3.3 DNA methylation profiling

Genome-wide DNA methylation profiling was performed using the Infinium MethylationEPIC BeadChip array (Illumina), covering over 850,000 CpG sites. DNA was extracted from FFPE tissue using the Maxwell^®^ RSC system (Promega), quantified with Qubit fluorometry (Life Technologies), and quality-checked according to the Infinium HD FFPE QC Assay protocol (Illumina). Bisulfite conversion was performed using the EZ DNA Methylation Kit (Zymo Research), followed by DNA restoration (Infinium HD FFPE Restore Kit, Illumina) and purification (DNA Clean & Concentrator Kit, Zymo Research). Whole-genome amplification, enzymatic fragmentation, and hybridization to the array were conducted following the Infinium HD Methylation Assay protocol (Illumina). Arrays were scanned using the Illumina iScan system. Signal intensity data were used to assess methylation profiles and genome-wide CNVs, including gains, losses, amplifications, and homozygous deletions. Data analysis was performed using the Heidelberg Brain Tumor Methylation Classifier (v12.5; https://www.molecularneuropathology.org/mnp). A calibrated classifier score ≥0.9 was required for definitive tumor class assignment; scores between 0.5 and 0.9 were considered for tumor family or subtype classification. All steps adhered to Illumina’s Infinium HD FFPE standards. Raw methylation data are available upon request.

## 4 Discussion

NEpTs harboring *EWSR1::PATZ1* fusions represent an exceptionally rare subgroup of primary brain neoplasms, currently unrecognized by the WHO classification system. Our case series is unique for several reasons. First, we describe two young patients (male and female) presenting with *EWSR1::PATZ1* fusion tumors in distinct neuroanatomical regions and exhibiting heterogeneous histopathological and molecular profiles. Second, we provide comprehensive methylation analysis, a feature underexplored in prior reports. Third, we identified a co-occurring *MUTYH* mutation, a finding not previously documented in this context, that cannot be determined as somatic or germline based on the molecular tests. Fourth, the therapeutic approaches varied between the two cases, providing valuable insights to inform future treatment recommendations for this entity. Fifth, both cases exhibited an intermediate prognosis compared to GBM, reinforcing the potential for prognostic stratification through the identification of this fusion. Finally, our cases, together with the insights obtained from the literature review, may provide a basis for the formal inclusion of these tumors in the WHO classification.

In this case series, we highlight the diagnostic challenges posed by *EWSR1::PATZ1*-fused tumors. Regarding the initial CNS imaging workup, our findings align with previous reports. For example, a study of seven cases analyzing cranial MRI features identified strong contrast enhancement, intralesional hemorrhage, cystic components, intraventricular extension (often associated with hydrocephalus), and calcifications as consistent imaging characteristics of these tumors (6). Both Case 1 and Case 2 exhibited key elements of these criteria, with marked contrast enhancement and a cystic component being shared imaging characteristics.

From a histopathological standpoint, Case 1 partially matched a pattern reported in a review of 14 literature cases and a pediatric tumor case report, featuring small round high-grade poorly differentiated cells with relatively scant eosinophilic cytoplasm, arranged in vascular pseudorosettes with significant stromal vascularization (7, 8). Case 2 exhibited notable diagnostic discordance. It was initially classified as an extraventricular neurocytoma by an external service based on its low-to-intermediate grade, small, monomorphic epithelioid cells with clear cell morphology – a diagnosis not previously associated with *EWSR1::PATZ1* fusions (5). However, upon reevaluation of the specimen from the recurrence, most of the characteristics were also present, supporting the diagnostic impression of NEpT. Notably, Ki-67 index diverged markedly (70% in high-grade Case 1 vs. 2-3% in low-grade Case 2) and the clinical impact of this finding is still unknown. While molecular diagnostics are indispensable in neuro-oncology, our data demonstrate that combined assessment through histopathology, immunohistochemistry, and cranial imaging can inform the diagnostic hypothesis of neuroepithelial tumors (NEpTs), especially in low-resource environments. Neuropathologist-driven evaluation enhances this strategy by mitigating morphological variability and promoting cost-effective use of molecular assays through targeted case selection for genomic and epigenomic profiling.

*EWSR1* and *PATZ1* are located in close proximity on chromosome 22. The *EWSR1::PATZ1* fusion arises from an intrachromosomal rearrangement between *EWSR1* (22q12.2) and *PATZ1* (22q12.2), resulting in an in-frame fusion that combines the N-terminal domain of *EWSR1* with the zinc finger domain of *PATZ1* (9). This molecular complexity justifies the necessity of NGS, particularly in young *IDH*-wildtype patients, to identify oncogenic drivers and actionable therapeutic targets (10). In Case 1, it is noteworthy that other hallmark characteristics of GBM, such as *EGFR* amplification, *TERT* promoter mutations, or gains of chromosome 7 with loss of chromosome 10, as well as other driver alterations in high-grade CNS neoplasms, were absent. In Case 2, the lack of *BRAF*, *FGFR1* or *MYB/MYBL* alterations – genes commonly implicated as sole drivers in low-grade NEpT – underscores *EWSR1::PATZ1* fusion role as the primary driver in these tumors (11).

The role of co-mutations also warrants further investigation. In sarcomas, *CDKN2A* alterations, reported in 71% of *EWSR1::PATZ1* fusion cases in a series of 11 patients, are associated with aggressive phenotypes and poorer outcomes (12). In Case 1, we identified a heterozygous *MUTYH* mutation (c.1103G>A, p.(Gly368Asp), G368D), whose somatic or germline status could not be determined by molecular testing, representing a novel finding in NEpT with *EWSR1::PATZ1* fusion. While germline *MUTYH* mutations are linked to diffuse midline pediatric gliomas, their somatic role in CNS carcinogenesis is yet to be fully elucidated. Interestingly, a single case of atypical central neurocytoma with *EWSR1::ATF1* fusion and *MUTYH* G382D mutation (detected via NGS) achieved ~3 years of PFS with conventional glioma therapy in a 13-year-old patient (13).

The *MUTYH c.1103*G*–>*A* (*p.Gly368Asp*) variant is a missense mutation associated with partial or complete loss of glycosylase activity, impairing the removal of mispaired adenines opposite 8-oxoguanine (8-oxoG) during base excision repair (BER). This deficiency compromises the cellular defense against *G:C* to *T:A* transversions, particularly under oxidative stress. In gliomas, temozolomide (TMZ) is a widely used alkylating agent that induces DNA damage primarily through methylation of guanine at the *O₆* position and promotes the generation of reactive oxygen species (ROS), further contributing to the accumulation of oxidative substrates such as 8-oxoG. In the absence of functional MUTYH, these oxidative lesions remain unrepaired, leading to replication errors, accumulation of somatic mutations, and progressive genomic instability. Over time, this process may favor the selection of resistant tumor subclones and contribute to the development of a hypermutated phenotype. This mutational profile has been reported in recurrent gliomas and is frequently associated with secondary alterations in mismatch repair genes, such as *MSH6* or *MSH2*, ultimately correlating with increased tumor aggressiveness and reduced responsiveness to standard therapies, including the Stupp protocol (14, 15).

Notably, Siegfried et al. analyzed methylation profiles in 7 of 40 BRAFV600-negative gangliogliomas and unexpectedly identified *EWSR1::PATZ1* fusions during molecular evaluation of a putative papillary ganglioglioma for *SLC44A1* (solute carrier family 44, member 1)::*PRKCA* (protein kinase C alpha) fusions. Their analysis revealed that these fusions define a distinct, well-differentiated methylation cluster, clearly segregated from other glioneuronal tumors. This molecular distinctiveness – coupled with the tumor’s ventricular localization and glioneuronal morphology – provides strong support for classifying EWSR1::PATZ1-fused tumors as a unique clinicopathological entity (16).

The prognostic and therapeutic implications of MGMT promoter methylation remain unclear in NEpTs harboring *EWSR1::PATZ1* fusions. In Case 1, the detection of *MGMT* promoter methylation in an *IDH*-wildtype, high-grade tumor prompted treatment with the Stupp protocol, a regimen typically used for GBM. The patient responded favorably to chemoradiotherapy, achieving a progression-free survival (PFS) of 14 months, exceeding the median PFS of 6.9 months reported in the phase III study by Stupp et al., *Radiotherapy plus Concomitant and Adjuvant Temozolomide for Glioblastoma* (PMID: 15758009) (17). In contrast, Case 2 experienced recurrence 8 months after the initial surgery. Key factors contributing to this outcome include the initial R1 resection and the absence of adjuvant therapy. Following a second R1 resection and subsequent adjuvant radiotherapy, the patient has remained disease-free for 11 months to date.

In our cohort, methylation profiling proved diagnostically decisive, aligning with findings from previous studies on sarcomas and CNS tumors (18, 19). Objective parameters, such as calibrated scores, may assist pathologists in the identification and stratification of these tumors, serving as a technical and reproducible benchmark for the development of a diagnostic algorithm for NEpTs. This molecular signature further substantiates the proposal to classify NEpTs as a distinct diagnostic entity. Its recognition as a core diagnostic element may inform future revisions of the WHO classification, supporting the broader integration of methylation profiling into CNS tumor diagnostics to enhance nosological precision. However, while methylation patterns are highly informative in distinguishing molecular subtypes, they may not fully capture tumor heterogeneity. Other epigenetic mechanisms, including histone modifications, chromatin remodeling, and non-coding RNA regulation, also influence tumor phenotypes. Epigenetic alterations evolve over time and across tumor regions, shaped by the microenvironment, selective therapy pressure, and clonal evolution. Therefore, relying solely on a single methylation snapshot for diagnosis may be insufficient, given the complexity of tumor biology (20, 21).

When comparing these results to those in low-grade gliomas, it is evident that the median PFS for grade 2 gliomas stands at 4 years, according to the RTOG 9802 (ClinicalTrials.gov, NCT00003375) study (22), whereas grade 3 gliomas demonstrate a median of 1.7 years, as shown in the RTOG 9402 (NCT00002569) (23), and 13.2 months, as documented in EORTC 26951 trial (NCT00002840) (24). Alhalabi et al. reported a median PFS of 144 months across 60 *PATZ1*-fused brain tumors but only 31 months (range: 6–144) for *EWSR1::PATZ1* fusion cases confirmed by RNA sequencing (25). While longer follow-up is needed, our findings position these tumors prognostically between GBM and low-grade gliomas.

*PATZ1* interacts with multiple genes implicated in apoptosis regulation, including *DR5, PUMA*, and *BCL-2* family members, offering actionable therapeutic targets. Recent drug screening in a GBM model with *MN1::PATZ1* fusion identified promising candidates: paclitaxel, D-actinomycin, volasertib (PLK1 inhibitor), navitoclax (BCL-2/BCL-XL/BCL-W inhibitor), and I-BET-151 (bromodomain inhibitor) (25, 26). The interplay between *PATZ1*, the tumor necrosis factor-related apoptosis-inducing ligand (*TRAIL*) pathway and TMZ offers a pivotal therapeutic axis. *TRAIL*, the natural ligand for apoptotic receptors, can trigger apoptosis independently of p53, which is mutated in up to 65% of GBM cases, partly contributing to TMZ resistance. TMZ may also act as a *TRAIL* “sensitizer”, overcoming resistance by upregulating death receptor expression and promoting significant caspase activation (27, 28). *PATZ1* functions as a transcriptional repressor that confers resistance to apoptosis, which is the primary antitumor mechanism of TMZ. In summary, targeting *PATZ1* could represent a novel therapeutic strategy to overcome TMZ resistance.

## 5 Conclusion

This report provides a comprehensive molecular and clinical characterization of high-grade and low-grade ultra-rare NEpTs harboring the *EWSR1::PATZ1* fusion, highlighting the role of methylation profiling as a valuable diagnostic tool. We present early treatment outcomes with glioma-based therapies and identify the first documented *MUTYH* mutation in these tumors, which may provide valuable insights into CNS tumorigenesis. Our findings reinforce the role of *EWSR1::PATZ1* as a driver alteration, defining a subset of tumors with distinct clinicopathological and molecular hallmarks, such as ventricular location, glioneuronal differentiation, and a unique methylation profile, with an intermediate prognosis. These characteristics strongly support its classification as an independent entity in future WHO revisions. Additionally, we encourage further research into targeted therapies that explore the downregulation of *PATZ1* expression, understanding its potential to serve as a therapeutic target in these tumors.

## Data Availability

The original contributions presented in the study are included in the article/supplementary material. Further inquiries can be directed to the corresponding author.
